# 2,3-Dimethyl-6-nitro-2*H*-indazole

**DOI:** 10.1107/S1600536809025410

**Published:** 2009-07-04

**Authors:** Yan Chen, Zheng Fang, Ping Wei

**Affiliations:** a College of Biotechnology and Pharmaceutical Engineering, Nanjing University of Technolgy, Xinmofan Road No. 5 Nanjing, Nanjing 210009, People’s Republic of China; b School of Pharmaceutical Sciences, Nanjing University of Technolgy, Xinmofan Road No. 5 Nanjing, Nanjing 210009, People’s Republic of China

## Abstract

In the mol­ecule of the title compound, C_9_H_9_N_3_O_2_, the indazole ring system is almost planar [maximum deviation = 0.019 (3) Å for the C atom bearing the nitro group]. In the crystal structure, inter­molecular C—H⋯O inter­actions link the mol­ecules into centrosymmetric dimers, forming *R*_2_^2^(18) ring motifs. Aromatic π–π contacts between indazole rings [centroid–centroid distances = 3.632 (1) and 3.705 (1) Å] may further stabilize the structure.

## Related literature

For a related structure, see: Xu *et al.* (1999 ▶). For bond-length data, see: Allen *et al.* (1987 ▶). For ring-motifs, see: Bernstein *et al.* (1995 ▶).
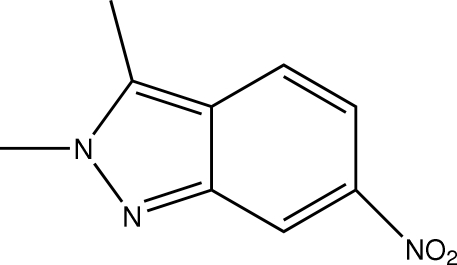


## Experimental

### 

#### Crystal data

C_9_H_9_N_3_O_2_
*M* *_r_* = 191.19
Triclinic, 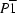

*a* = 6.5800 (13) Å
*b* = 7.2050 (14) Å
*c* = 10.752 (2) Å
α = 75.07 (3)°
β = 74.67 (3)°
γ = 66.73 (3)°
*V* = 444.81 (19) Å^3^
*Z* = 2
Mo *K*α radiation
μ = 0.11 mm^−1^
*T* = 294 K
0.30 × 0.20 × 0.10 mm

#### Data collection

Enraf–Nonius CAD-4 diffractometer
Absorption correction: ψ scan (North *et al.*, 1968 ▶) *T*_min_ = 0.969, *T*_max_ = 0.990
1756 measured reflections
1606 independent reflections
1292 reflections with *I* > 2σ(*I*)
*R*_int_ = 0.031
3 standard reflections frequency: 120 min intensity decay: 1%

#### Refinement

*R*[*F* ^2^ > 2σ(*F* ^2^)] = 0.054
*wR*(*F* ^2^) = 0.154
*S* = 1.00
1606 reflections
129 parameters
H-atom parameters constrained
Δρ_max_ = 0.32 e Å^−3^
Δρ_min_ = −0.25 e Å^−3^

### 

Data collection: *CAD-4 Software* (Enraf–Nonius, 1989 ▶); cell refinement: *CAD-4 Software*; data reduction: *XCAD4* (Harms & Wocadlo, 1995 ▶); program(s) used to solve structure: *SHELXS97* (Sheldrick, 2008 ▶); program(s) used to refine structure: *SHELXL97* (Sheldrick, 2008 ▶); molecular graphics: *ORTEP-3 for Windows* (Farrugia, 1997 ▶) and *PLATON* (Spek, 2009 ▶); software used to prepare material for publication: *SHELXL97* and *PLATON*.

## Supplementary Material

Crystal structure: contains datablocks global, I. DOI: 10.1107/S1600536809025410/hk2724sup1.cif

Structure factors: contains datablocks I. DOI: 10.1107/S1600536809025410/hk2724Isup2.hkl

Additional supplementary materials:  crystallographic information; 3D view; checkCIF report

## Figures and Tables

**Table 1:** Hydrogen-bond geometry (Å, °)

*D*—H⋯*A*	*D*—H	H⋯*A*	*D*⋯*A*	*D*—H⋯*A*
C1—H1*A*⋯O2^i^	0.96	2.58	3.533 (4)	171

Symmetry code: (i) 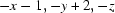.

**Fig. 1.**
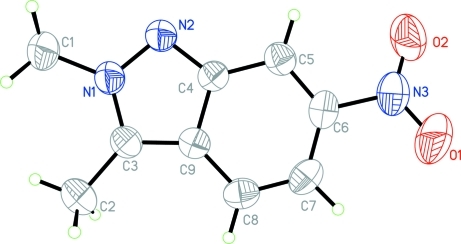
The molecular structure of the title molecule, with the atom-numbering scheme. Displacement ellipsoids are drawn at the 30% probability level.

**Fig. 2.**
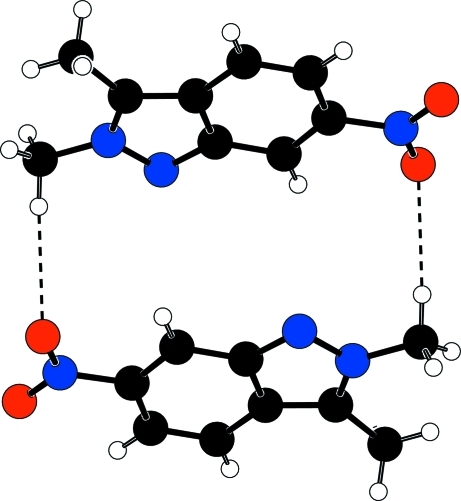
A partial packing diagram of the title compound. Hydrogen bonds are shown as dashed lines.

## supplementary crystallographic information

### Comment

Some derivatives of indazole are important chemical materials. We report herein the crystal structure of the title compound.

In the molecule of the title compound (Fig 1), the bond lengths (Allen *et al.*, 1987) and angles are within normal ranges. Rings A (N1/N2/C3/C4/C9) and B (C4-C9) are, of course, planar and the dihedral angle between them is A/B = 0.80 (3)°. The indazole ring system is planar with a maximum deviation of -0.019 (3) Å for atom C6. Atoms O1, O2, N3, C1 and C2 are 0.024 (3), -0.124 (3), -0.038 (3), 0.003 (3) and -0.056 (3) Å away from the plane of the indazole ring system, respectively.

In the crystal structure, weak intermolecular C-H···O interactions (Table 1) link the molecules into centrosymmetric dimers forming *R*_2_^2^(18) ring motifs (Bernstein *et al.*, 1995) (Fig. 2), in which they may be effective in the stabilization of the structure. The π–π contacts between the indazole rings, Cg1—Cg2^i^ and Cg2—Cg2^ii^ [symmetry codes: (i) 2 - x, 2 - y, -z, (ii) 2 - x, 1 - y, -z, where Cg1 and Cg2 are centroids of the rings A (N1/N2/C3/C4/C9) and B (C4-C9), respectively] may further stabilize the structure, with centroid-centroid distances of 3.632 (1) and 3.705 (1) Å, respectively.

### Experimental

For the preparation of the title compound, metallic sodium (3.22 g) was dissolved in regurgitant 2-propanol (140 ml). Then, the solution was added to 3-methyl-6-nitro-1*H*-indazole (13 g) and iodomethane (30 g) was added in small portions. The mixture was refluxed for 5 h. The suspension was cooled to room temperature, filtered and washed with 2-propanol to give yellow solid (yield; 12 g) (Xu *et al.*, 1999). Crystals suitable for X-ray analysis were obtained by slow evaporation of a methanol solution.

### Refinement

H atoms were positioned geometrically, with C-H = 0.93 and 0.96 Å for aromatic and methyl H, respectively, and constrained to ride on their parent atoms, with U_iso_(H) = xU_eq_(C), where x = 1.5 for methyl H and x = 1.2 for aromatic H atoms.

### Crystal data

**Table:**

C_9_H_9_N_3_O_2_	*Z* = 2
*M**_r_* = 191.19	*F*(000) = 200
Triclinic, *P*1	*D*_x_ = 1.427 Mg m^−^^3^
Hall symbol: -P 1	Mo *K*α radiation, λ = 0.71073 Å
*a* = 6.5800 (13) Å	Cell parameters from 25 reflections
*b* = 7.2050 (14) Å	θ = 9–13°
*c* = 10.752 (2) Å	µ = 0.11 mm^−^^1^
α = 75.07 (3)°	*T* = 294 K
β = 74.67 (3)°	Block, colorless
γ = 66.73 (3)°	0.30 × 0.20 × 0.10 mm
*V* = 444.81 (19) Å^3^	

### Data collection

**Table:**

Enraf–Nonius CAD-4 diffractometer	1292 reflections with *I* > 2σ(*I*)
Radiation source: fine-focus sealed tube	*R*_int_ = 0.031
graphite	θ_max_ = 25.3°, θ_min_ = 2.0°
ω/2θ scans	*h* = 0→7
Absorption correction: ψ scan (North *et al.*, 1968)	*k* = −7→8
*T*_min_ = 0.969, *T*_max_ = 0.990	*l* = −12→12
1756 measured reflections	3 standard reflections every 120 min
1606 independent reflections	intensity decay: 1%

### Refinement

**Table:**

Refinement on *F*^2^	Secondary atom site location: difference Fourier map
Least-squares matrix: full	Hydrogen site location: inferred from neighbouring sites
*R*[*F*^2^ > 2σ(*F*^2^)] = 0.054	H-atom parameters constrained
*wR*(*F*^2^) = 0.154	*w* = 1/[σ^2^(*F*_o_^2^) + (0.08*P*)^2^ + 0.235*P*] where *P* = (*F*_o_^2^ + 2*F*_c_^2^)/3
*S* = 1.00	(Δ/σ)_max_ < 0.001
1606 reflections	Δρ_max_ = 0.32 e Å^−^^3^
129 parameters	Δρ_min_ = −0.25 e Å^−^^3^
0 restraints	Extinction correction: *SHELXL97* (Sheldrick, 2008), Fc^*^=kFc[1+0.001xFc^2^λ^3^/sin(2θ)]^-1/4^
Primary atom site location: structure-invariant direct methods	Extinction coefficient: 0.059 (12)

### Special details

**Table:**

Geometry. All e.s.d.'s (except the e.s.d. in the dihedral angle between two l.s. planes) are estimated using the full covariance matrix. The cell e.s.d.'s are taken into account individually in the estimation of e.s.d.'s in distances, angles and torsion angles; correlations between e.s.d.'s in cell parameters are only used when they are defined by crystal symmetry. An approximate (isotropic) treatment of cell e.s.d.'s is used for estimating e.s.d.'s involving l.s. planes.
Refinement. Refinement of *F*^2^ against ALL reflections. The weighted *R*-factor *wR* and goodness of fit *S* are based on *F*^2^, conventional *R*-factors *R* are based on *F*, with *F* set to zero for negative *F*^2^. The threshold expression of *F*^2^ > σ(*F*^2^) is used only for calculating *R*-factors(gt) *etc*. and is not relevant to the choice of reflections for refinement. *R*-factors based on *F*^2^ are statistically about twice as large as those based on *F*, and *R*- factors based on ALL data will be even larger.

### Fractional atomic coordinates and isotropic or equivalent isotropic displacement parameters (Å^2^)

**Table:**

	*x*	*y*	*z*	*U*_iso_*/*U*_eq_	
O1	0.2155 (5)	0.7495 (5)	−0.3831 (2)	0.1020 (9)	
O2	−0.1339 (4)	0.7845 (4)	−0.33747 (19)	0.0803 (7)	
N1	−0.2315 (3)	0.7731 (3)	0.25319 (18)	0.0469 (5)	
N2	−0.3309 (3)	0.7997 (3)	0.15107 (18)	0.0479 (5)	
N3	0.0392 (4)	0.7648 (3)	−0.3051 (2)	0.0611 (6)	
C1	−0.3707 (5)	0.7880 (5)	0.3829 (2)	0.0660 (8)	
H1A	−0.5144	0.8941	0.3758	0.099*	
H1B	−0.2979	0.8202	0.4368	0.099*	
H1C	−0.3915	0.6594	0.4218	0.099*	
C2	0.1367 (5)	0.6977 (4)	0.3176 (3)	0.0601 (7)	
H2B	0.0456	0.7087	0.4032	0.090*	
H2C	0.2098	0.7971	0.2929	0.090*	
H2D	0.2482	0.5625	0.3186	0.090*	
C3	−0.0080 (4)	0.7362 (3)	0.2216 (2)	0.0432 (6)	
C4	−0.1582 (3)	0.7777 (3)	0.0489 (2)	0.0390 (5)	
C5	−0.1637 (4)	0.7888 (3)	−0.0830 (2)	0.0446 (6)	
H5A	−0.2966	0.8160	−0.1108	0.054*	
C6	0.0376 (4)	0.7573 (3)	−0.1678 (2)	0.0462 (6)	
C7	0.2431 (4)	0.7224 (4)	−0.1315 (2)	0.0517 (6)	
H7A	0.3741	0.7057	−0.1943	0.062*	
C8	0.2482 (4)	0.7134 (3)	−0.0049 (2)	0.0470 (6)	
H8A	0.3819	0.6910	0.0204	0.056*	
C9	0.0474 (3)	0.7388 (3)	0.0874 (2)	0.0387 (5)	

### Atomic displacement parameters (Å^2^)

**Table:**

	*U*^11^	*U*^22^	*U*^33^	*U*^12^	*U*^13^	*U*^23^
O1	0.1021 (19)	0.150 (2)	0.0491 (12)	−0.0512 (17)	0.0174 (12)	−0.0311 (13)
O2	0.1002 (17)	0.0986 (16)	0.0521 (12)	−0.0371 (13)	−0.0217 (11)	−0.0183 (11)
N1	0.0480 (11)	0.0573 (12)	0.0389 (10)	−0.0226 (9)	−0.0063 (8)	−0.0089 (8)
N2	0.0436 (11)	0.0606 (12)	0.0427 (11)	−0.0218 (9)	−0.0069 (8)	−0.0098 (9)
N3	0.0804 (16)	0.0572 (13)	0.0453 (12)	−0.0255 (11)	−0.0051 (12)	−0.0129 (10)
C1	0.0621 (17)	0.095 (2)	0.0422 (14)	−0.0311 (15)	0.0000 (12)	−0.0173 (13)
C2	0.0642 (16)	0.0669 (17)	0.0564 (15)	−0.0235 (13)	−0.0231 (13)	−0.0092 (12)
C3	0.0475 (13)	0.0402 (12)	0.0459 (12)	−0.0177 (9)	−0.0112 (10)	−0.0077 (9)
C4	0.0400 (11)	0.0369 (11)	0.0427 (12)	−0.0162 (9)	−0.0058 (9)	−0.0085 (9)
C5	0.0501 (13)	0.0444 (12)	0.0442 (13)	−0.0207 (10)	−0.0117 (10)	−0.0061 (9)
C6	0.0611 (14)	0.0384 (12)	0.0389 (12)	−0.0202 (10)	−0.0034 (10)	−0.0079 (9)
C7	0.0465 (13)	0.0485 (13)	0.0523 (14)	−0.0148 (10)	0.0048 (10)	−0.0128 (11)
C8	0.0405 (12)	0.0423 (12)	0.0579 (14)	−0.0137 (9)	−0.0079 (10)	−0.0104 (10)
C9	0.0426 (12)	0.0306 (10)	0.0448 (12)	−0.0135 (8)	−0.0098 (9)	−0.0070 (8)

### Geometric parameters (Å, °)

**Table:**

O1—N3	1.222 (3)	C2—H2C	0.9600
O2—N3	1.223 (3)	C2—H2D	0.9600
N1—N2	1.357 (3)	C3—C9	1.389 (3)
N1—C1	1.456 (3)	C4—C5	1.409 (3)
N1—C3	1.350 (3)	C4—C9	1.420 (3)
N2—C4	1.346 (3)	C5—C6	1.366 (3)
N3—C6	1.460 (3)	C5—H5A	0.9300
C1—H1A	0.9600	C6—C7	1.416 (4)
C1—H1B	0.9600	C7—C8	1.354 (3)
C1—H1C	0.9600	C7—H7A	0.9300
C2—C3	1.487 (3)	C8—C9	1.405 (3)
C2—H2B	0.9600	C8—H8A	0.9300
			
N2—N1—C1	118.6 (2)	N1—C3—C2	124.3 (2)
C3—N1—N2	114.84 (19)	C9—C3—C2	130.3 (2)
C3—N1—C1	126.6 (2)	N2—C4—C5	127.8 (2)
C4—N2—N1	102.99 (17)	N2—C4—C9	111.81 (19)
O1—N3—O2	122.6 (2)	C5—C4—C9	120.4 (2)
O1—N3—C6	118.2 (2)	C6—C5—C4	116.1 (2)
O2—N3—C6	119.2 (2)	C6—C5—H5A	121.9
N1—C1—H1A	109.5	C4—C5—H5A	121.9
N1—C1—H1B	109.5	C5—C6—C7	124.2 (2)
H1A—C1—H1B	109.5	C5—C6—N3	117.7 (2)
N1—C1—H1C	109.5	C7—C6—N3	118.1 (2)
H1A—C1—H1C	109.5	C8—C7—C6	119.7 (2)
H1B—C1—H1C	109.5	C8—C7—H7A	120.1
C3—C2—H2B	109.5	C6—C7—H7A	120.1
C3—C2—H2C	109.5	C7—C8—C9	118.6 (2)
H2B—C2—H2C	109.5	C7—C8—H8A	120.7
C3—C2—H2D	109.5	C9—C8—H8A	120.7
H2B—C2—H2D	109.5	C3—C9—C8	134.1 (2)
H2C—C2—H2D	109.5	C3—C9—C4	105.00 (19)
N1—C3—C9	105.36 (19)	C8—C9—C4	120.9 (2)
			
C1—N1—N2—C4	179.6 (2)	C2—C3—C9—C8	−2.1 (4)
C3—N1—N2—C4	0.1 (2)	N2—C4—C5—C6	−178.9 (2)
N2—N1—C3—C2	−178.6 (2)	C9—C4—C5—C6	0.9 (3)
N2—N1—C3—C9	0.2 (2)	N2—C4—C9—C3	0.4 (2)
C1—N1—C3—C2	1.9 (4)	N2—C4—C9—C8	−179.25 (18)
C1—N1—C3—C9	−179.3 (2)	C5—C4—C9—C3	−179.41 (19)
N1—N2—C4—C5	179.5 (2)	C5—C4—C9—C8	0.9 (3)
N1—N2—C4—C9	−0.3 (2)	C4—C5—C6—C7	−2.2 (3)
O1—N3—C6—C5	175.6 (2)	C4—C5—C6—N3	179.20 (19)
O1—N3—C6—C7	−3.1 (3)	C5—C6—C7—C8	1.8 (4)
O2—N3—C6—C5	−5.0 (3)	N3—C6—C7—C8	−179.7 (2)
O2—N3—C6—C7	176.4 (2)	C6—C7—C8—C9	0.2 (3)
N1—C3—C9—C4	−0.4 (2)	C7—C8—C9—C3	179.0 (2)
N1—C3—C9—C8	179.3 (2)	C7—C8—C9—C4	−1.4 (3)
C2—C3—C9—C4	178.3 (2)		

### Hydrogen-bond geometry (Å, °)

**Table:**

*D*—H···*A*	*D*—H	H···*A*	*D*···*A*	*D*—H···*A*
C1—H1A···O2^i^	0.96	2.58	3.533 (4)	171

Symmetry codes: (i) −*x*−1, −*y*+2, −*z*.

## References

[bb1] Allen, F. H., Kennard, O., Watson, D. G., Brammer, L., Orpen, A. G. & Taylor, R. (1987). *J. Chem. Soc. Perkin Trans. 2*, pp. S1–19.

[bb2] Bernstein, J., Davis, R. E., Shimoni, L. & Chang, N.-L. (1995). *Angew. Chem. Int. Ed. Engl.* **34**, 1555-1573.

[bb3] Enraf–Nonius (1989). *CAD-4 Software* Enraf–Nonius, Delft, The Netherlands.

[bb4] Farrugia, L. J. (1997). *J. Appl. Cryst.* **30**, 565.

[bb5] Harms, K. & Wocadlo, S. (1995). *XCAD4* University of Marburg, Germany.

[bb6] North, A. C. T., Phillips, D. C. & Mathews, F. S. (1968). *Acta Cryst.* A**24**, 351–359.

[bb7] Sheldrick, G. M. (2008). *Acta Cryst.* A**64**, 112–122. 10.1107/S0108767307043930 18156677

[bb8] Spek, A. L. (2009). *Acta Cryst.* D**65**, 148–155. 10.1107/S090744490804362X PMC2631630 19171970

[bb9] Xu, B.-C., Deng, F. & Wang, H.-Z. (1999). *Speciality Petro. Chem.* pp. 18–20.

